# Role of NRF2 in protection of the gastrointestinal tract against oxidative stress

**DOI:** 10.3164/jcbn.17-139

**Published:** 2018-05-03

**Authors:** Akinori Yanaka

**Affiliations:** 1 Hitachi Medical Education and Research Center, University of Tsukuba Hospital, Division of Gastroenterology, Faculty of Medicine, University of Tsukuba, 1-1-1 Tennodai, Tsukuba 305-8575, Japan

**Keywords:** nuclear factor erythroid 2-related factor 2, oxidative stress, gastrointestinal tract, Kelch-like erythroid cell-derived protein with CNC homology-associated protein 1, antioxidant systems

## Abstract

The gastrointestinal tract is exposed to a variety of noxious factors, such as *Helicobacter pylori*, nonsteroidal anti-inflammatory drugs, gastric acid, ischemia-reperfusion, and mental stresses. Theses stressors generate free radicals within gastrointestinal tissues, causing organ injury and functional disturbance. Although the gastrointestinal tract can withstand such oxidative stresses to some extent by enhancing its antioxidant system via nuclear factor erythroid 2-related factor 2-Kelch-like erythroid cell-derived protein with CNC homology-associated protein 1-mediated pathways, acute or chronic exposure to oxidative stress can cause several gastrointestinal tract disorders, such as inflammation, ulcers, cancers, and various functional disturbances. Recent studies have demonstrated that some natural compounds and drugs can upregulate the nuclear factor erythroid 2-related factor 2-mediated antioxidant system, ameliorating or preventing these disorders. Although these compounds may be useful as chemopreventive agents, sufficient evidence for their clinical efficacy has not yet been provided. In addition, it is important to note that excessive nuclear factor erythroid 2-related factor 2 stimulation can be harmful to human health, especially from the standpoint of tumor biology.

## Oxidative Stress Causes Cell and Tissue Injury

Oxidative stress involves the exposure of cells and/or tissues to reactive oxygen species (ROS) generated by various intrinsic and extrinsic factors. Exposure to acute or chronic oxidative stress causes cellular damage and impairs the normal physiological functions of various organs, causing a variety of diseases, such as acute organ failure, chronic degenerative diseases, and cancers. However, cells can withstand oxidative stress by activating systems that scavenge free radicals, protecting them from critical damage. The nuclear factor erythroid 2-related factor 2 (NRF2)-Kelch-like erythroid cell-derived protein with CNC homology-associated protein 1 (KEAP1) pathway, originally discovered by Itoh *et al.*,^(1)^ is an important scavenger system that protects cells against oxidative stress.^(2,3)^ In this review, I first provide some examples of gastrointestinal (GI) disorders caused by oxidative stress. Second, I outline the role of NRF2 in protecting GI organs from oxidative stress-induced diseases. Third, I discuss natural and synthetic chemical agents that enhance NRF2-mediated protection against oxidative GI disorders. Finally, I mention the negative aspects of NRF2 stimulation, especially from the viewpoint of tumor biology.

## GI Disorders Caused by Oxidative Stress

### Reflux esophagitis

The healthy gastric mucosa is protected from back-diffusion of luminal gastric acid by the impermeability of the apical membrane tight junctions of the gastric epithelial cells (GECs).^(4)^ In contrast, the esophageal mucosa is relatively susceptible to gastric luminal acid.^(5)^ Thus, reflux of gastric luminal acid into the esophageal lumen readily causes esophageal mucosal injury, manifested clinically as reflux esophagitis. It has been reported that H^+^ not only injures epithelial cells, but also causes free radical generation in their mitochondria,^(6–8)^ exacerbating acid-induced injury and inflammation.^(9)^ This suggests that free radicals generated by luminal H^+^ contribute to the pathophysiology of reflux esophagitis.

### *Helicobacter pylori*-associated gastroduodenal disease

Although a number of epidemiological studies have shown a strong association between *Helicobacter pylori* (*H. pylori*) infection and various gastric diseases^(10)^ the exact mechanisms by which *H. pylori* infection causes gastric mucosal injury were not well understood until GI investigators focused on its role in oxidative stress.^(11,12)^ *H. pylori* colonization in GECs causes the accumulation of neutrophils and macrophages within the gastric mucosa, resulting in the generation of superoxide anion (O_2_^−^) and nitric oxide (NO).^(13–15)^ These conditions are histopathologically recognized as *H. pylori*-induced gastritis. Continuous release of free radicals from mucosal neutrophils accumulated within the *H. pylori*-infected gastric mucosa gradually results in GEC apoptosis, leading to gastric atrophy and intestinal metaplasia. In some cases, long-term infection with *H. pylori* can cause various neoplasms, such as hyperplastic polyps, adenomas, and carcinomas. In addition to causing the accumulation of neutrophils in the gastric mucosa, *H. pylori* infection releases CagA protein into the cytoplasm of GECs, resulting in free radical generation in the mitochondria of CagA-infected cells.^(16)^ Free radicals generated by *H. pylori* infection, together with gastric luminal acid, degrade the tight junction structures of the gastric and duodenal epithelia, resulting in enhanced back-diffusion of luminal acid into GECs. As a result, ulcers develop in the gastroduodenal mucosae.^(17,18)^

### Nonsteroidal anti-inflammatory drug (NSAID)-induced GI ulcers

Recent global trends show increased ingestion of aspirin and/or NSAIDs with the aging of the human population.^(10)^ Aspirin and/or NSAID intake frequently induces ulcers, erosions, and bleeding in the GI tract. It has been reported that aspirin and/or NSAIDs generate free radicals by several mechanisms.^(19)^ NSAIDs enhance neutrophil adhesion to endothelial cells, inducing free radical generation in the endothelial cells.^(20–22)^ In the upper GI tract, in addition to free radical generation, gastric acid is necessary for NSAID-induced ulcer formation,^(23)^ since most NSAID-induced ulcers are mitigated or prevented by potent acid inhibitors, such as proton pump inhibitors (PPI)^(24)^ and potassium-competitive acid blockers.^(25)^ However, in the small intestine, acid inhibitors do not mitigate, but sometimes exacerbate aspirin-induced ulcer development.^(26)^ Aspirin induces small intestinal ulceration by generating free radicals,^(27–29)^ possibly in the mitochondria of small intestinal cells.^(30)^ Dysbiosis induced by potent acid inhibitors may play a role in NSAID-induced injury to the small intestine.^(26,31)^

### Inflammatory bowel disease (IBD)

The exact mechanisms of IBD have not yet been clarified. However, numerous studies have shown that excessive amounts of free radicals are generated in IBD patients by various factors, such as autoimmune abnormalities, changes in microbiota, and recent changes to Western style diets. In ulcerative colitis (UC), neutrophils accumulate within the colonic mucosa and generate microabscesses, which cause mucosal inflammation and ulcers.^(32–35)^ Prolonged inflammation causes continuous exposure to free radicals, thereby increasing the risk of developing colon cancer.^(36,37)^ In Crohn’s disease, intraluminal antigens derived from the diet and/or microbiota activate mucosal macrophages, which produce inflammatory cytokines, such as tumor necrosis factor-α (TNF-α), interleukin (IL)-1β, IL-6, and IL-8. These cytokines induce infiltration of polymorphonuclear leukocytes and mononuclear cells into the GI tissues, which causes overproduction of free radicals. This results in an imbalance between oxidative stress and the antioxidant systems, thereby causing transmural inflammation, ulcers, and fibrosis in the GI tract.^(38–40)^

### Functional GI disorders

Recent studies have provided evidence that functional GI disorders, such as gastric motility disease^(12)^ and irritable bowel syndrome (IBS),^(41)^ are also associated with oxidative stress. It has been suggested that the disturbance of gastric motility is caused by damage to the intramural smooth muscle cells and by dysfunction of the neuromuscular junction, which is composed of enteric nerves and interstitial cells of Cajal (ICC). A number of previous studies in experimental animals has shown that dysfunction of these components is observed during sepsis, ischemia/reperfusion stress, and diabetes mellitus. Details in the pathogenesis of GI motility disorders have been provided in a previous review.^(12)^ In addition, we have recently shown that daily intake of sulforaphane (SFN)-rich broccoli sprouts improves defecation in human patients with chronic constipation, which may also indicate that NRF2 stimulation by dietary intake of SFN strengthens antioxidative defense systems, thereby preserving ICC-dependent GI motility.^(42)^ It has been reported that mild inflammation is associated with the pathogenesis of IBS, since serum levels of inflammatory cytokines are increased in patients with IBS compared with those in healthy subjects. In addition, serum cytokine levels correlate well with IBS symptom scores, indicating that oxidative stress may play some role in the pathogenesis of IBS.^(41)^

## Mechanisms by which NRF2 Protects the GI Tract against Oxidative Stress

**Fig. 1:**
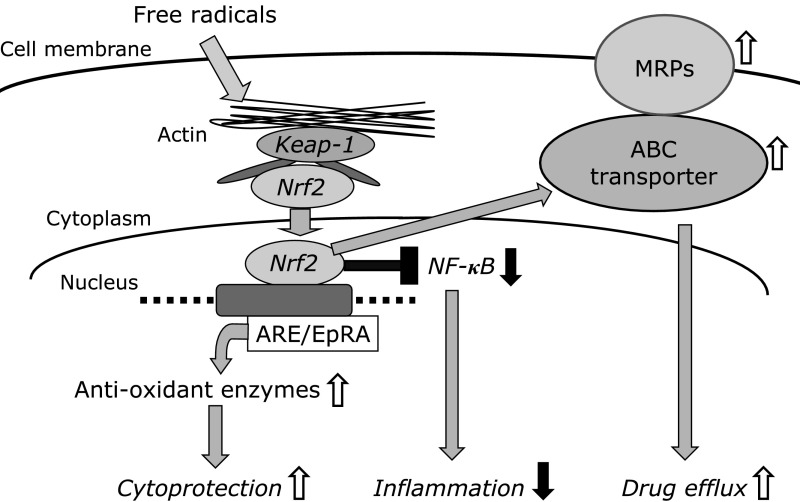
Mechanisms by which NRF2 affords cytoprotection against oxidative stress.

NRF2 protects cells from oxidative stress and subsequent inflammation through several mechanisms. In this chapter, three major and different mechanisms, illustrated in Fig. 1, are discussed.

### Upregulation of antioxidant and xenobiotic enzymes

Oxidative stress causes severe damage to GECs. However, it also dissociates the inactive form of NRF2 from KEAP1 in the cytoplasm, and induces its translocation into the nucleus. Once in the nucleus, NRF2 binds to antioxidant response elements and upregulates the expression of antioxidant enzymes, thereby strengthening the cell’s ability to neutralize several types of free radicals.^(43)^ NRF2 also contributes to the preservation of the fine structures of tight junctions, and maintains epithelial polarity, which is essential for mucosal protection of the upper GI tract against gastric luminal acid.^(44,45)^ NRF2 also upregulates xenobiotic-metabolizing enzymes such as glutathione-*S*-transferase and UDP-glucosidase, which are mainly expressed in the small intestine.^(46,47)^

### Amelioration of inflammation by downregulation of nuclear factor κB (NFκB)

It has been shown that NRF2 not only enhances antioxidant enzyme activity, but also upregulates inhibitor of κB (IκB) and downregulates NFκB, thereby inhibiting proinflammatory signaling and mitigating inflammation.^(48)^ Mitigation of the inflammatory response contributes to the protection of the GI mucosa against oxidative injury. An anti-inflammatory role of NRF2 has been reported in experimentally induced uremic rats, in which inhibition of NRF2 function exacerbates intestinal inflammation and disrupts epithelial barrier function.^(49)^

### Stimulation of ATP-binding cassette (ABC) transporters and multidrug resistance-associated protein 2 (MRP2)

Recent studies have demonstrated NRF2-dependent induction of ABC transporters under oxidative stress,^(50)^ indicating that NRF2 contributes to the efflux of various substances by ABC transporter upregulation.^(51)^ With respect to bile acid transporters, NRF2 regulates MRP2 and the bile salt export pump in human hepatocytes, which excrete bile acids into bile.^(52)^ These NRF2-mediated stimulations of normal hepatic or intestinal transport contribute to organ protection from oxidative injury. In contrast to normal healthy cells, however, NRF2-dependent induction of these transporters causes drug resistance in cancer cells, suggesting a negative role of NRF2 in clinical cancer chemotherapy, as discussed below.

## Natural and Synthetic Chemical Compounds that Enhance NRF2-Dependent Protection of the GI Tract against Oxidative Stress

There are a number of chemical compounds that can upregulate the NRF2-dependent antioxidant system, protecting cells and tissues from oxidative injury. Some of these substances are natural compounds found in plants and animals, which can be ingested in the diet. Others are drugs previously developed for other functions. All of these compounds can stimulate NRF2 signaling in cells *in vitro* and/or in experimental animals *in vivo*. Although some of these compounds may be useful as chemopreventive agents, sufficient evidence of their clinical efficacy has not yet been provided. A summary of the previous basic and clinical reports regarding the efficacy of these compounds on oxidative stress-induced injuries is provided in Table 1.

### Natural compounds in food

A variety of natural compounds in plants and animals, such as isothiocyanates, polyphenols, and carotenoids, possess anti-oxidant properties and can thereby mitigate oxidative stress. It has been suggested that daily intake of food containing these compounds ameliorates inflammation, and retards the progression of atherosclerosis, cancer development, diabetes mellitus, degenerative diseases, and aging in humans.^(53–55)^ The mechanisms by which these compounds exhibit anti-oxidant properties have been studied extensively. For example, polyphenols and carotenoids exhibit anti-oxidant activity by functioning as ROS scavengers. Furthermore, recent studies have revealed that some types of isothiocyanates, polyphenols, and carotenoids enhance anti-oxidant activity via nrf2-keap1-mediated mechanisms in response to oxidative stress. Although some studies have shown the clinical efficacy of these compounds,^(53–55)^ sufficient evidence on the clinical efficacy of most of the other compounds has not been well documented. In this chapter, the nrf2-mediated antioxidant effects of natural food components on the GI tract are mainly discussed.

#### Isothiocyanates

##### 1) Sulforaphane (SFN)

SFN is an isothiocyanate compound generated from glucosinolates, which are rich in cruciferous vegetables such as broccoli, cabbage, and radishes, and especially broccoli sprouts.^(46)^ SFN has been shown to prevent not only a variety of cancers, but also cardiovascular diseases, neurodegenerative diseases, diabetes, and aging.^(53)^ We have previously shown that SFN stimulates the expression of NRF2-dependent antioxidant enzymes both *in vitro* and *in vivo*, and protects cells and tissues from *H. pylori*- and NSAID-induced oxidative injury.^(28,29,56,57)^ In some of these studies, we also found that SFN inhibits *H. pylori* activity in the gastric mucosa,^(56)^ and anaerobic enteric bacteria in the small intestinal mucosa.^(28)^ Furthermore, our clinical trials have shown that dietary intake of sulforaphane glucosinolate (SGS), a precursor of SFN, stimulates antioxidant enzymes in the human GI tract, and ameliorates gastric inflammation in *H. pylori*-infected subjects.^(56)^ We have also shown that dietary intake of SGS reduces *H. pylori* levels in the gastric lumen, thereby providing chemoprotection against gastric cancer.^(56)^ Details regarding the basic mechanisms by which SFN protects against cancer can be found in Fuentes *et al.*^(58)^ In addition, our recent study has shown that dietary intake of SGS improves defecation in human subjects, presumably by upregulating antioxidant enzyme activities.^(42)^ Taken together, we believe that SFN is a promising compound in the protection of the GI tract from oxidative injury.

##### 2) *Brassica* plant-derived isothiocyanates other than SFN

Several types of isothiocyanates, such as allyl isothiocyanate (AITC) and authentic 6-(methylsulfinyl)hexyl isothiocyanate (6-HITC) are found in cruciferous vegetables and are especially high in wasabi.^(59,60)^ Both AITC and 6-HITC have been shown to activate NRF2.^(60–62)^ For example, an *in vitro* study in rat liver epithelial cells demonstrated that 6-HITC potently stimulates antioxidant response element transcription, inducing phase 2 enzymes, and that these effects of 6-HITC were abrogated in NRF2-deficient cells.^(60,61)^ A recent study in human volunteers showed that daily intake of food levels of AITC does not cause DNA strand breaks, estimated by measuring urinary levels of 8-hydroxy-2'-deoxyguanosine (8-OHdG).^(62)^ However, no data have been reported so far, from either *in vivo* animal studies or human studies, which demonstrate the protective effects of these compounds on the GI tract against oxidative stress.

#### Polyphenols

Polyphenols are secondary metabolites of plants, and are considered to enhance the defense system against human chronic diseases induced by prolonged oxidative stress. Recent studies have shown that dietary intake of polyphenols contributes to many types of chronic diseases induced by oxidative stress.^(54)^ Hydroxyl groups linked with the benzene bond in many types of polyphenols reduce oxidative stress not only by oxidizing themselves, but also by chelating metals such as copper and iron, which oxidize the cells.^(54)^ Details of these classic mechanisms and their association with various types of clinical diseases are described elsewhere.^(54)^ This review focuses on the nrf2-dependent protection of the GI tract induced by some polyphenols during oxidative stress.

##### 1) Curcumin

Curcumin is a polyphenol found at a high level in turmeric, which is used as a spice, food colorant, and traditional herbal medicine.^(63)^ *In vitro* studies in rat renal epithelial cells^(61)^ and mouse macrophages^(64)^ have shown that both curcumin and its synthetic analog, dimethoxycurcumin, activate the expression of heme oxygenase-1 (HO-1) by stimulating the binding of NRF2 to its antioxidant response element.^(64,65)^ Previous clinical trials on curcumin have shown its effectiveness in mitigating symptoms in patients with IBS.^(66,67)^ A clinical trial in IBS patients has shown that daily intake of curcumin in combination with fennel essential oil for 30 days improved the symptom scores and quality of life for IBS patients.^(66)^ Another clinical intervention study in UC patients has shown that induction with NCB-02 (curcumin) enema ameliorates symptoms in patients with mild-to-moderate distal UC.^(67)^

##### 2) Catechin

Catechins are phytochemicals that are highly enriched in tea.^(68)^ Basic studies have shown that catechin protects against ketoprofen-induced oxidative damage of the gastric mucosa by upregulating NRF2 *in vitro* and *in vivo*.^(69)^ Epigallocatechin gallate upregulates NRF2 by disabling KEAP1, preventing diabetic nephropathy.^(70)^ Several epidemiological studies have suggested that catechin intake may reduce the risk of human GI disorders. For example, higher phenolic acid concentrations in the plasma and urine of men consuming green or black tea have demonstrated potential chemopreventive properties for colon cancer.^(71)^ A prospective cohort comparison of flavonoid treatment in patients with resected colorectal cancer to prevent recurrence has shown that sustained long-term treatment with a flavonoid mixture can reduce the recurrence rate of colon neoplasia in patients with resected colon cancer.^(72)^ Observational studies conducted in Finland have shown that high flavonoid intake was associated with low risk of pancreatic cancer in male smokers.^(73)^ Only one intervention trial has shown a protective effect of green tea polyphenols in liver cancer prevention among high-risk individuals.^(74)^

##### 3) Quercetin

Quercetin is a flavonoid that is highly enriched in citrus fruits and onions.^(75)^ An *in vitro* study in liver cancer-derived HepG2 cells has shown that quercetin modulates redox signaling by upregulation of NRF2 expression and downregulation of NFκB and cyclooxygenase-2 (COX-2), thereby mitigating oxidative injury induced by a cytotoxic agent, ochratoxin A.^(76)^ A recent *in vivo* study in rats has demonstrated that pretreatment with quercetin mitigates indomethacin-induced GI injury via upregulation of NRF2 and downregulation of NFκB, supporting the protective role of quercetin against NSAID-induced oxidative injury in the GI tract.^(77)^ Although some previous clinical trials have shown that daily intake of quercetin improves biomarkers of metabolic syndromes,^(78,79)^ no clinical data have been reported regarding the protective effect of quercetin in the GI tract during oxidative stress in human subjects.

##### 4) Resveratrol

Resveratrol (trans-3,4',5-trihydroxystilbene), a polyphenolic phytoalexin, is rich in grapes and other fruits and plants.^(80,81)^ The protective effects of resveratrol against oxidative stress involve not only direct neutralization of reactive oxygen species, but also upregulation of NRF2-dependent antioxidant enzymes during oxidative stress.^(81,82)^ A recent study showed that resveratrol enhances heat stress-induced upregulation of antioxidant enzymes via NRF2-dependent mechanisms, and protects quail hepatocytes from oxidative stress induced by high ambient temperatures.^(83)^ Since resveratrol possesses anti-inflammatory and antioxidant activity, and it inhibits multiple immune responses in colonic mucosa, resveratrol may be useful as a treatment option for IBD.^(84)^ A recent clinical trial demonstrated that 6 weeks of supplementation with 500 mg resveratrol decreases plasma levels of TNF-α and mitigates clinical colitis in patients with UC,^(85)^ supporting the possibility of resveratrol as a treatment option for UC in the future.

#### Carotenoids

Carotenoids are organic pigments that are produced by plants and animals. Daily intake of carotenoids reduces the risk of chronic diseases induced by oxidative stress.^(55)^ It has been well known that several types of carotenoids attenuate oxidative stress by scavenging free radicals.^(55)^ Details of these mechanisms and their association with various types of clinical diseases have been described elsewhere.^(55)^ This review addresses the roles of lycopene and astaxanthin in the nrf2-dependent protection of the GI tract against oxidative stress.

##### 1) Lycopene

Lycopene is a bright red carotene enriched in tomatoes and other red fruits and vegetables, such as red carrots and watermelons.^(86)^ An *in vitro* study using human bronchial epithelial cells has shown that enzymatic metabolites of lycopene induce NRF2-mediated expression of phase II antioxidant enzymes.^(87)^ Recent *in vivo* studies have shown that lycopene not only ameliorates atrazine-induced oxidative damage in the adrenal cortex of male rats by activating the NRF2/HO-1 pathway,^(88)^ but also attenuates oxidative stress-induced neuroinflammation and cognitive impairment via the NRF2/NFκB transcriptional pathway.^(89)^ Although some prior clinical trials showed that administration of lycopene improves the profiles of oxidative biomarkers in humans,^(90,91)^ no clinical trials have yet shown the clinical efficacy of lycopene in treating human GI disorders.

##### 2) Astaxanthin

Astaxanthin is a carotenoid enriched in shrimp and salmon.^(92)^ Astaxanthin intervention ameliorates cyclophosphamide-induced oxidative stress, DNA damage, and early hepatocarcinogenesis in rats, via the actions of NRF2, p53, p38, and phase-II enzymes.^(93)^ Astaxanthin and β-carotene can prevent *H. pylori*-induced gastric inflammation.^(94)^ Astaxanthin and omega-3 fatty acids protect against oxidative stress via the NRF2-ARE pathway, both individually and in combination.^(95)^ Although some clinical studies have shown that intake of astaxanthin improves biomarkers of systemic oxidative stress, the effects on GI diseases have not been extensively studied.^(96,97)^ However, a recent clinical trial has demonstrated that higher dose of astaxanthin, 40 mg/day, reduced reflux symptoms in patients with gastroesophageal reflux disease (GERD), but had no curative effects on functional dyspepsia.^(98)^ Further studies are required regarding the clinical efficacy of astaxanthin to treat oxidative stress-induced GI disorders.

### Drugs & Hormones

#### Lansoprazole (LPZ)

LPZ is a PPI, originally developed in Japan.^(99)^ LPZ not only inhibits gastric acid secretion,^(99)^ but also shows anti-inflammatory effects.^(100)^ An *in vitro* study using rat gastric mucosal cells demonstrated that LPZ, at a concentration of 1–100 µM, which approximates doses used to inhibit gastric acid secretion, upregulates HO-1 expression through an NRF2-KEAP1-meditated mechanism.^(100)^ Another *in vitro* study showed that LPZ, at concentrations of 10–100 µM, inhibits mitochondrial superoxide production and cellular lipid peroxidation induced by indomethacin in GECs, supporting the possibility that LPZ enhances cellular defenses against oxidative stress.^(101)^ However, an *in vivo* study in rat stomach demonstrated that LPZ failed to stimulate NRF2 expression, although it strongly inhibited indomethacin-induced gastric ulcers, suggesting that the acid inhibitory effect of LPZ is more important than the role of NRF2, at least in stomach.^(102)^ In contrast, another *in vivo* study using rat small intestines have shown that LPZ, but not omeprazole, prevented indomethacin-induced small intestinal ulceration through induction of HO-1, suggesting that NRF2-mediated protection plays an important role against indomethacin-induced oxidative stress in the absence of luminal acid.^(103)^ Although numerous clinical studies have shown that LPZ prevents NSAID/aspirin-induced injury in the upper GI tract, no clinical studies to date have clearly demonstrated that the protective effects of LPZ on the GI tract are mediated by NRF2-dependent mechanisms. Further studies are required to assess the role of LPZ.

#### Ursodeoxycholic acid (UDCA)

UDCA, a drug known to protect human liver function, stimulates NRF2-mediated hepatocellular transport, detoxification, and antioxidant stress systems in mice.^(104)^ Studies in diabetic mice have shown that UDCA inhibits the expression of proinflammatory cytokines and foam cell formation via upregulation of ABC transporters, thereby blocking atherosclerosis ^(105)^. In patients with Barrett’s esophagus and in Barrett’s cell lines, UDCA increases expression of antioxidant enzymes and prevents DNA damage by bile acids.^(106)^ All of these findings strongly suggest that NRF2-mediated stimulation of normal hepatic or intestinal transport by UDCA contributes to the protection of the liver and small intestine from oxidative injury. However, sufficient evidence for the protective role of UDCA in oxidative GI injury has not yet been provided. Thus, future clinical studies are required to address this possibility.

#### Sofalcone

Sofalcone, originally developed as a gastric mucosal protective agent, has been shown to increase vascular endothelial growth factor via the NRF2-HO-1 pathway in GECs.^(107)^ It also has been shown that sofalcone increases mucus gel thickness and mucosal blood flow in the gastric mucosa.^(108)^ Although previous clinical trials of sofalcone have shown that it promotes human gastric ulcer healing,^(109)^ no studies have shown that this effect is mediated by an antioxidant system. Although the clinical efficacy of sofalcone against gastroduodenal ulcers is far less than that observed with potent acid inhibitors, such as PPIs or PCABs, the effects on small intestinal ulcers may be different. Therefore, it seems worthwhile to examine the effect of sofalcone on NSAID/aspirin-induced small intestinal injury in both animal models and human patients.

#### Ghrelin

Ghrelin, a gut-brain peptide hormone secreted from the gastric corpus as well as from brain tissues, was originally discovered as a gut hormone and plays an important role in appetite regulation.^(110–112)^ Previous studies have demonstrated that ghrelin protects the GI mucosa from ethanol-induced injuries, effects mediated by crosstalk between endogenous prostaglandins and NO.^(112,113)^ More recent *in vivo* studies in rats have shown that ghrelin upregulates HO-1 expression and protects gastric mucosa against indomethacin-induced injury^(114)^ and ischemia/reperfusion injury,^(115)^ suggesting the involvement of NRF2-mediated induction of the antioxidant system in the protection afforded by ghrelin. Another recent study from China showed that ghrelin protects lung tissues from oxidative injury induced by paraquat, and the authors demonstrated upregulation of NRF2 by ghrelin, supporting the possibility that ghrelin affords organ protection against oxidative stress by NRF2-mediated mechanism.^(116)^ Furthermore, a recent clinical trial on relamorelin, a ghrelin receptor agonist, showed that it mitigates vomiting and accelerates gastric emptying in patients with diabetic gastroparesis, indicating that ghrelin may be useful as a therapeutic drug for GI disorders induced by various types of oxidative stress.^(117)^ Further studies are required to assess this possibility.

#### Melatonin

Melatonin, known as *N*-acetyl-5-methoxytryptamine, is a hormone produced by the pineal gland, which regulates sleep and wakefulness.^(118)^ Several studies have shown that melatonin enhances antioxidant properties, thereby protecting cells from oxidative stress by upregulating the NRF2-mediated antioxidant system.^(119)^ A recent *in vivo* mouse study has shown that melatonin not only prevents dextran sodium sulfate (DSS)-induced colitis, but also prevents the formation of colitis-associated colonic carcinoma induced by a chemical carcinogen; both effects are mediated by NRF2-dependent mechanisms.^(120)^ Several clinical trials have been conducted regarding the therapeutic effects of melatonin on oxidative stress-induced GI disorders. For example, treatment with melatonin, 3 mg/day, for 4 to 8 weeks caused significant improvement in GERD-related symptoms, although the effects were slightly smaller than those induced by omeprazole, 40 mg/day.^(121)^ However, melatonin treatment in combination with omeprazole demonstrated enhanced efficacy compared with that observed with omeprazole alone, indicating that melatonin may be useful as a therapeutic drug for PPI-resistant GERD symptoms.^(121)^ Another clinical study showed that treatment with melatonin, 8 mg/day, for 6 months significantly improved symptoms of IBS in postmenopausal women.^(122)^ Furthermore, another clinical trial has shown that treatment with melatonin, 5 mg/day, in combination with mesalazine, 2 g/day, for 12 months, significantly reduced the relapse rate in patients with UC in remission.^(123)^ All three studies have provided supportive data to indicate that melatonin may be useful as a therapeutic drug for GERD, IBS, and UC in the future. Further studies are required to confirm these findings, and to assess the safety of long-term use of melatonin in human subjects.

## Negative Aspects of NRF2 Activation: NRF2 as a Double-Edged Sword

Numerous prior studies have revealed that NRF2 plays an important role in protecting the GI tract against various oxidative stresses, thereby contributing to chemoprevention against GI cancers. However, it is important to note the negative aspects of NRF2, with special emphasis on its effects on cancer cells.^(124,125)^ In experimental mice, mutation of the gene encoding NRF2 or overexpression of NRF2 by KEAP1 knockout enhances cancer cell proliferation.^(126,127)^ Stimulation of ABC transporters and multidrug resistance protein 2 (MDR2) by NRF2 facilitates the clearance of anticancer drugs, which in turn induces chemoresistance in cancer cells and promotes tumor growth.^(128,129)^ Such cases have been reported clinically for several types of cancers. However, no report has shown that long-term intake of NRF2-stimulating compounds causes similar conditions. However, clinicians should be wary of this possibility, especially when administering these agents to cancer patients.

## Figures and Tables

**Table 1:** Representative studies on the effects of various compounds on NRF2-mediated protection of gastrointestinal tract and liver against oxidative stresses

		Basic Study			Clinical Study	
		*In vitro*	*In vivo*		Observational study	Intervention study
Isothiocyanates	Sulforaphane	28, 29	28, 29, 56			42 (Constipation) 56 (*H. pylori*-gastritis)
	Alyl-isothicyanate	60	61			

Polyphenols	Curcumin	64, 65				66 (IBS), 67 (UC)
	Catechin	69	69, 70		71, 72, 73	
	Quercetin	76	77		78, 79	
	Resveratrol	81, 82	83			85 (UC)

Carotenoids	Lycopene	87	88, 89			
	Astaxanthin	93, 95	93			98 (FD)

Drugs	Lansoprazole	100, 101	102 (No effect), 103			
	UDCA	104	105			106 (Barret esophagus)
	Sofalcon	107	108			109 (Gastric uler)

Hormones	Ghrelin		112, 113, 114, 115, 116			117 (Diabetic gastroparesis)
	Melatonin	119	120			121 (GERD), 122 (IBS) 123 (UC)

Corresponding reference numbers in this paper are indicated in the Table. IBS, irriable bowel syndrome; UC, ulcerative colitis; GERD, gastroesophageal reflux disease; FD, functional dyspepsia.
